# Computer-assisted femoral head reduction osteotomies: an approach for anatomic reconstruction of severely deformed Legg-Calvé-Perthes hips. A pilot study of six patients

**DOI:** 10.1186/s12891-020-03789-y

**Published:** 2020-11-18

**Authors:** P. Fürnstahl, F. A. Casari, J. Ackermann, M. Marcon, M. Leunig, R. Ganz

**Affiliations:** 1 Research in Orthopedic Computer Science (ROCS), Balgrist University Hospital, University of Zurich, Zurich, Switzerland; 2 Orthopedic Department, Balgrist University Hospital, Zurich, Switzerland; 3 Institute for Orthopedic Biomechanics, ETH Zurich, Zurich, Switzerland; 4 Radiology Department, Balgrist University Hospital, Zurich, Switzerland; 5 Schulthess Clinic, Zurich, Switzerland; 6 Faculty of Medicine, University of Berne, Berne, Switzerland

**Keywords:** Femoral head reduction osteotomy, Computer-assisted surgery, Three-dimensional preoperative planning, Patient-specific instruments

## Abstract

**Background:**

Legg–Calvé–Perthes (LCP) is a common orthopedic childhood disease that causes a deformity of the femoral head and to an adaptive deformity of the acetabulum. The altered joint biomechanics can result in early joint degeneration that requires total hip arthroplasty. In 2002, Ganz et al. introduced the femoral head reduction osteotomy (FHRO) as a direct joint-preserving treatment. The procedure remains one of the most challenging in hip surgery. Computer-based 3D preoperative planning and patient-specific navigation instruments have been successfully used to reduce technical complexity in other anatomies. The purpose of this study was to report the first results in the treatment of 6 patients to investigate whether our approach is feasible and safe.

**Methods:**

In this retrospective pilot study, 6 LCP patients were treated with FHRO in multiple centers between May 2017 and June 2019. Based on patient-specific 3D-models of the hips, the surgeries were simulated in a step-wise fashion. Patient-specific instruments tailored for FHRO were designed, 3D-printed and used in the surgeries for navigating the osteotomies. The results were assessed radiographically [diameter index, sphericity index, Stulberg classification, extrusion index, LCE-, Tönnis-, CCD-angle and Shenton line] and the time and costs were recorded. Radiologic values were tested for normal distribution using the Shapiro–Wilk test and for significance using Wilcoxon signed-rank test.

**Results:**

The sphericity index improved postoperatively by 20% (*p* = 0.028). The postoperative diameter of the femoral head differed by only 1.8% (*p* = 0.043) from the contralateral side and Stulberg grading improved from poor coxarthrosis outcome to good outcome (*p* = 0.026). All patients underwent acetabular reorientation by periacetabular osteotomy. The average time (in minutes) for preliminary analysis, computer simulation and patient-specific instrument design was 63 (±48), 156 (±64) and 105 (±68.5), respectively.

**Conclusion:**

The clinical feasibility of our approach to FHRO has been demonstrated. The results showed significant improvement compared to the preoperative situation. All operations were performed by experienced surgeons; nevertheless, three complications occurred, showing that FHRO remains one of the most complex hip surgeries even with computer assistance. However, none of the complications were directly related to the simulation or the navigation technique.

## Background

**Fig. 1:**
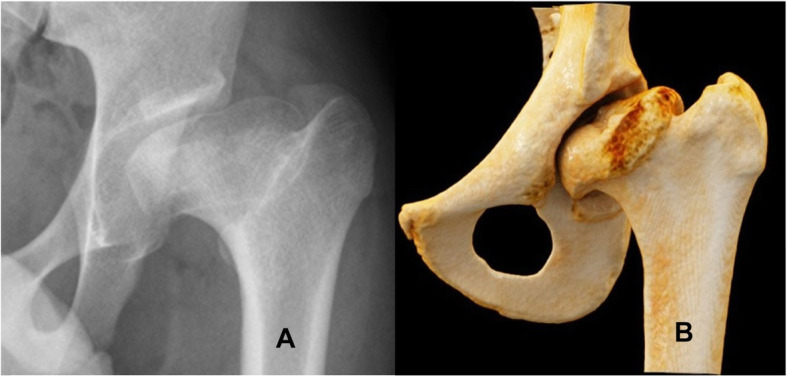
Radiographs (**a**) and volumetric rendering (**b**) of a hip affected by LCP disease. The new contour of the head resembles a mushroom with a short neck and has a high-riding trochanter

Legg–Calvé–Perthes (LCP) is an orthopedic childhood disease caused by a disturbance in the blood supply to the femoral head. With a lifetime risk of about 1 in 1200 children, LCP disease can be considered as one of the most common hip disorders in young children [1]. The pathology presents itself at the age of 4 to 8 years, but it may take 5 to 10 years until the full deformity has manifested [1–4]. Avascular necrosis of the femoral head develops in the first phase of the pathology. Later, the head of the femur progressively deforms as a result of fatigue fractures caused by repetitive forces acting on the joint during daily activities. The new contour of the head resembles a mushroom with a central dent [5] or a saddle [6] that has a significant three-dimensional (3D) component [7]. The horizontal diameter is extra-large (coxa magna), the neck is short and the greater trochanter is high-riding (Fig. 1). The dysmorphic head induces adaptive changes of the acetabulum in the form of a secondary acetabular dysplasia [8], leading to impaired hip function and pain [9] due to intra- and extracapsular impingement [7], hinged abduction [10, 11] and early joint degeneration [8, 9].

Conservative treatment possibilities are load restriction, physiotherapy and orthoses; however, these treatments are ineffective [4]. Possible surgical options after healed LCP include adductor tenotomy [12], osteochondroplasty [13], valgus-extension intertrochanteric osteotomy [13, 14] and acetabular osteotomy [15, 16], but these approaches do not correct of the actual bone deformity. In one-third of the patients, pain and stiffness reach unbearable levels due to early joint degeneration. Femoral head reduction osteotomy (FHRO) remains the only joint-preserving surgical treatment option [5, 7, 17–20].

**Fig. 2:**
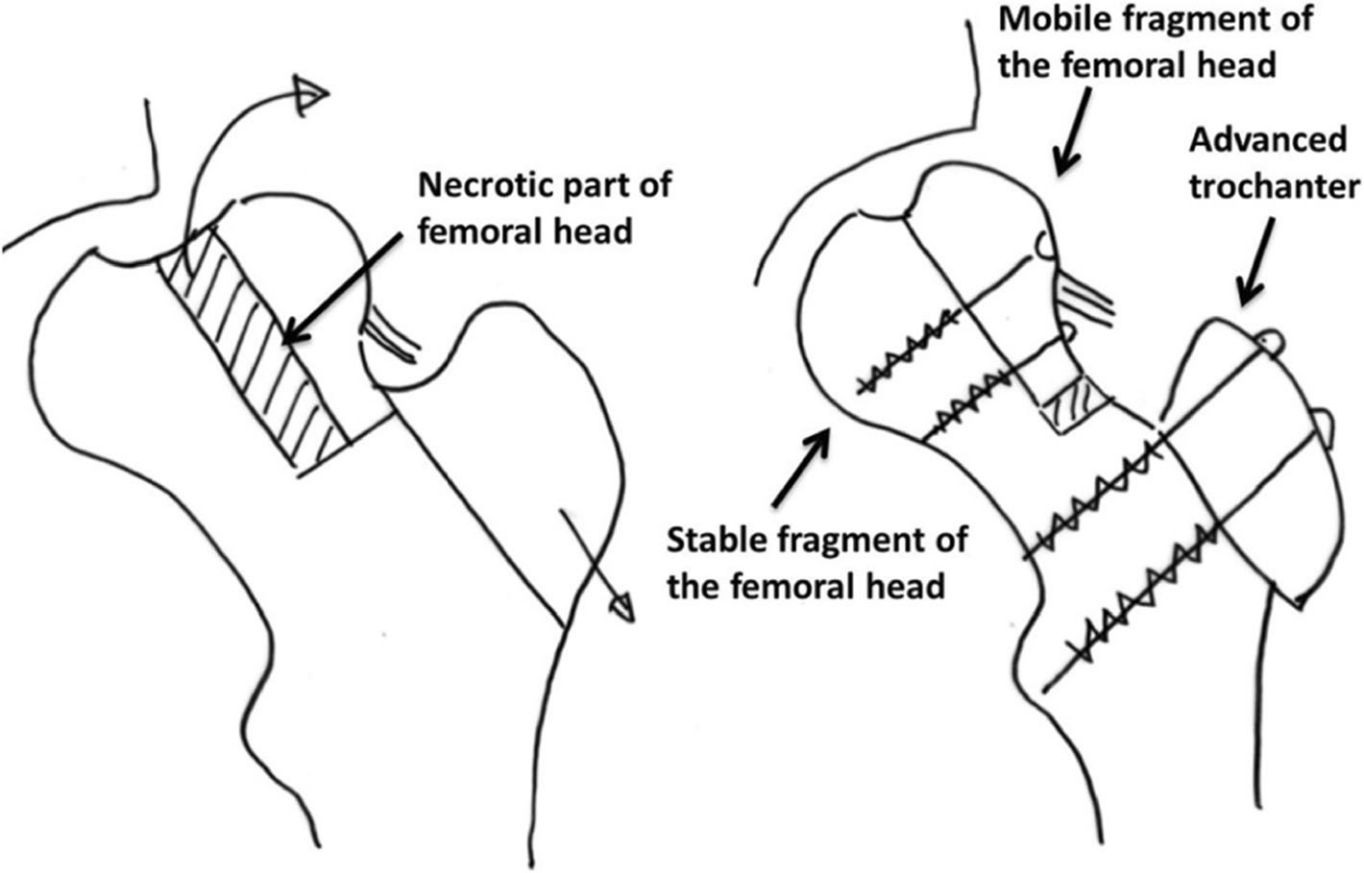
Modified form of Fig. 9 in the work by Ganz et al. [17]

Ganz performed the first FHRO in 2002 and published first results in 2009 (Fig. 2) [17]. The procedure aims at restoring the sphericity of the femoral head as much as possible. The osteotomies separate the head into a mobile lateral fragment, a central necrotic and a stable medial part [5, 17, 18]. The pathologically extended central part is resected and the lateral fragment is carefully reduced. In the majority of cases, a concurrent reorientation of the acetabulum by periacetabular osteotomy (PAO) [21] is needed to restore the joint containment and stability [7].

Careful and detailed preoperative planning of the procedure is necessary [13], but state-of-the-art planning is limited to conventional imaging using biplanar radiographs, MRI and CT [7, 20]. Surgeons have to rely on simplified two-dimensional measures to decide on the size of the resection and the direction of the osteotomies. For this reason, a final decision can only be made intraoperatively and the surgeon has no choice other than to perform the most difficult femoral head osteotomies in a freehand fashion [7].

We have developed a new technique that combines computer simulation for preoperative planning and additive manufacturing for surgical navigation with patient-specific instruments, with the goal to perform FHRO in a more controlled manner. The purpose of this study was to report the first results in the treatment of 6 patients to investigate whether our approach is safe and feasible.

## Materials and methods

In this retrospective multicenter pilot study, 7 patients [age range: 9–18 years] underwent our proposed approach for surgical FHRO treatment between May 2017 and October 2019. The surgeries were performed at different centers by four senior hip surgeons who had previous experience in FHRO surgeries. One out of 7 patients was excluded from this study because the parents/legal guardian did not provide informed consent. Ethical approval was obtained by the ethical committee of the Canton of Zurich. Three patients were male and the other three were female. Inclusion criteria were pain and restricted hip motion, severe deformity of the femoral head and intact peripheral cartilage with central necrosis. Preoperative computer simulation of the surgery was performed for each patient and patient-specific instruments (PSI) for surgical navigation were designed and manufactured.

### Preoperative computer simulation

**Fig. 3:**
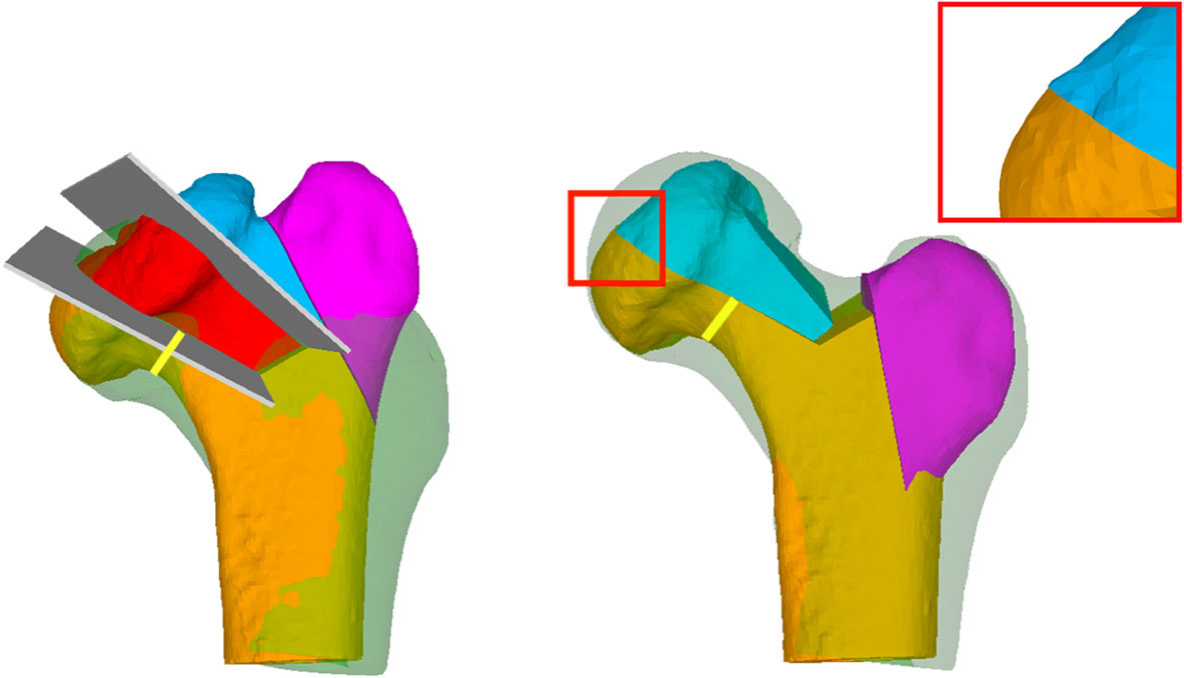
Preoperative computer simulation of the FHRO. The greater trochanter is shown in purple, the mobile fragment in blue, the necrotic part in red, and the stable fragment in orange. The yellow line represents the remaining femoral neck thickness and the shadow contour the template. **a** Preoperative pathological femur. **b** Postoperative femur. The red square visualizes the contact zone between the fragments

CT scans of the patients᾽ hips were obtained in a supine position and anterior-posterior [AP] hip orientation according to a specifically designed protocol [MyOsteotomy CT protocols, Medacta International, Castel San Pietro, Switzerland]. The CT scans were acquired with an axial resolution of 1 mm slice thickness using a Philips Brilliance 40 CT device [Philips Healthcare, Best, the Netherlands]. The data was imported into a commercial image processing software [Mimics Medical, Version 19; Materialise, Leuven, Belgium] and the bone anatomy was segmented from the surrounding soft tissues by applying global intensity-based thresholding and region-growing. 3D triangular surface models of the femur and the pelvis were generated from the segmented images using the Marching Cube algorithm [22]. These models were imported into the in-house developed preoperative planning software CASPA [Computer-Assisted Surgical Planning Application, Version 5.29] to simulate the FHRO surgeries (Fig. 3). The mirrored models of the healthy contralateral sides (Fig. 3, shadow contour) were used to approximate the pre-morbid femoral heads and served as remodeling templates in the simulation. In the case of a pathological contralateral side, a geometric sphere was used as a template instead. The sphere was manually centered in the mechanical joint center of the hip and resized until it covered the healthy portion of the femoral head.

The definition of the femoral head osteotomy planes is the most important step in the preoperative planning (Fig. 3a, grey planes). These planes implicitly define the resection of the necrotic part (Fig. 3a, red wedge), the degree of head sphericity, the residual articular step-off between the contact surfaces of the fragments (Fig. 3b, red square), and the size of the remaining neck pillar (Fig. 3, yellow line). The locations of the osteotomies are constrained by the medial and lateral retinacular blood vessels feeding the femoral head.

**Fig. 4:**
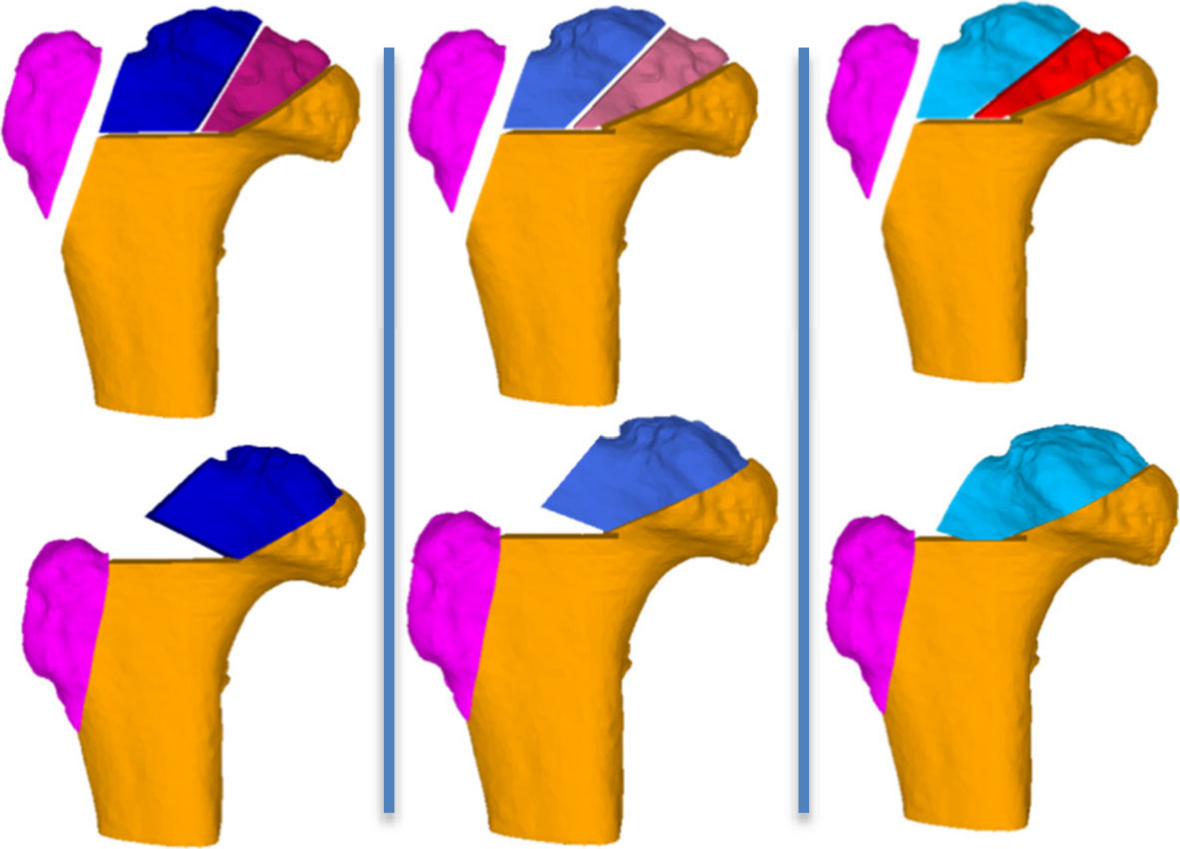
Iterative fine-tuning of the osteotomy planes (top row) and reductions (bottom row) is required until the optimal solution for the FHRO can be found. Each column represents one simulated planning solution. The right-most solution was implemented in the surgery

**Fig. 5:**
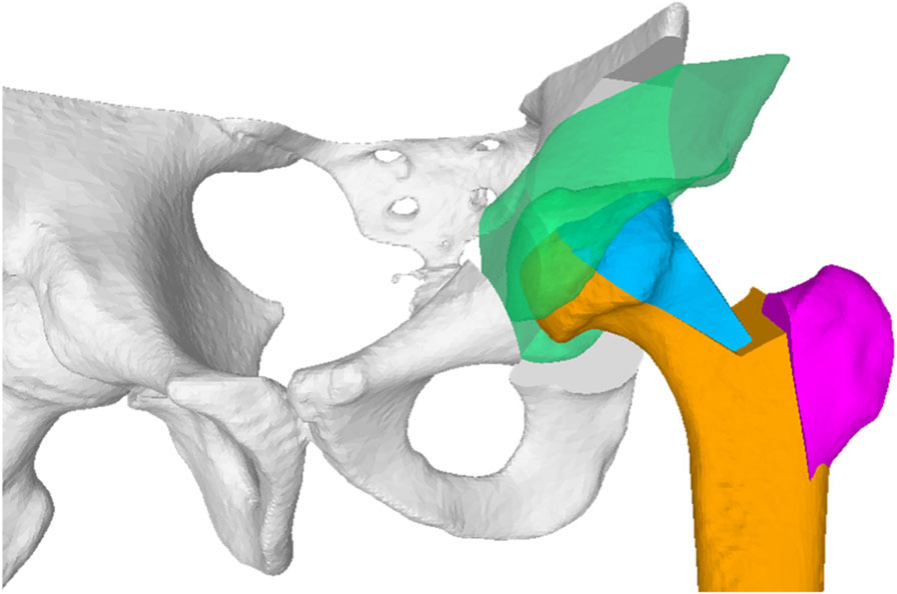
Simulation of a combined FHRO and PAO. The reduced fragments of the femur and acetabulum are shown in blue and green, respectively

The first osteotomy was defined along the lateral end of the necrotic area to create the mobile fragment (Fig. 3, blue). The reduction of the mobile fragment was simulated by applying 3D rotations and translations such that the sphericity, the articular step-off, and the neck pillar size are optimized. The intersectional volume of the mobile fragment in its reduced position and the stable part was then used to determine the orientation of the second osteotomy plane, which provides the definition for the 3D wedge that is to be resected. Iterative refinement of the orientation of the osteotomy plane was required in each case until the optimal strategy was determined (Fig. 4). After each FHRO simulation, the congruency and fitting of the reshaped head into the acetabulum was assessed in order to reveal the necessity and extent of the additional PAO (Fig. 5).

### PSI design

**Fig. 6:**
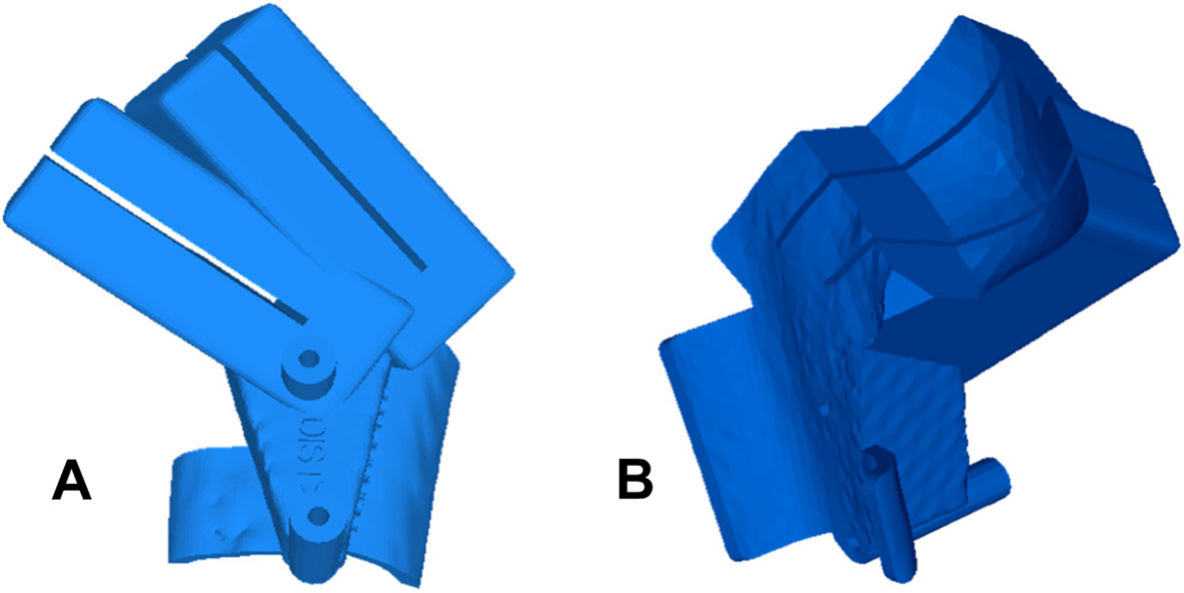
The proposed PSI design for the navigation of FHRO. **a** The PSI contains two cutting slits for guiding the blade of the surgical saw and two drill sleeves for temporary fixation of the PSI with surgical pins. **b** The undersurface of the PSI is shaped as a negative of the patient’s bone surface

**Fig. 7:**
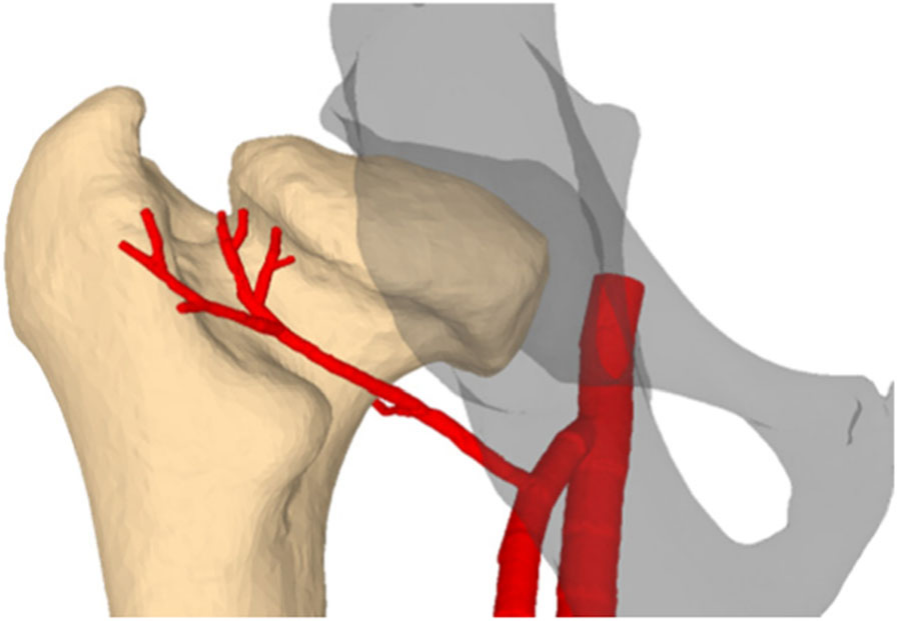
Posterior view of an LCP-affected hip with a schematic 3D model of the medial circumflex femoral artery (red)

PSI refers to a surgical navigation concept in which the cutting, drilling, and reduction instruments are computer-designed and matched with the preoperative simulation of the surgery. The undersurfaces of the instruments are shaped as the negatives of the bone anatomy such that the tools can later be placed exactly in the planned positions on the bone (Fig. 6). PSI as navigation tools for corrective osteotomies were first introduced for the treatment of complex malunions of the forearm bones [23–25]. We have adopted the PSI approach by designing new instruments tailored to the anatomy of the proximal femur and the FHRO. The main challenge was to design a PSI that can be placed on the proximal femur without compromising the vascular supply at the infero-medial curve of the femoral neck (Fig. 7). The remaining footprint of the anterior bone surface on which the PSI can be placed is small and the surface relief of the bone is insufficiently pronounced to provide sufficient guide stability. For this reason, medial and lateral hooks were integrated into the base block of the PSI in order to improve its stability. An offset of 4 mm was integrated into the portion of the cartilaginous part of the head. Two drill sleeves of Ø 2.6 mm were designed to allow the temporary fixation of the PSI on the bone with surgical pins. The PSI also consisted of two cutting slits into which the blade of the surgical saw could be inserted and aligned according to the planned osteotomy planes. The PSI were manufactured as CE-conformed medical products by an industrial partner [Medacta International, Castel San Pietro, Switzerland] using biocompatible polyamide [P2200; EOS GmbH, Germany] and a selective laser sintering device [Formiga P395/ P396/ P100, EOS GmbH, Krailling, Germany]. Before surgery, autoclave sterilization was performed in the surgical centers.

### Surgical technique

The patient was positioned in the lateral decubitus position. The pathologic hip was accessed via the surgical hip dislocation approach [26]. The medial femoral circumflex artery was secured in the form of a pediculated periosteal flap [6, 17]. For the dissection of the retinacular flap, the stable part of the trochanter was resected piecemeal down to the level of the neck and the periosteum was carefully dissected, allowing free access to the lateral and posterior neck bone. For the FHRO, the medial retinaculum was left attached to the calcar area [17].

**Fig. 8:**
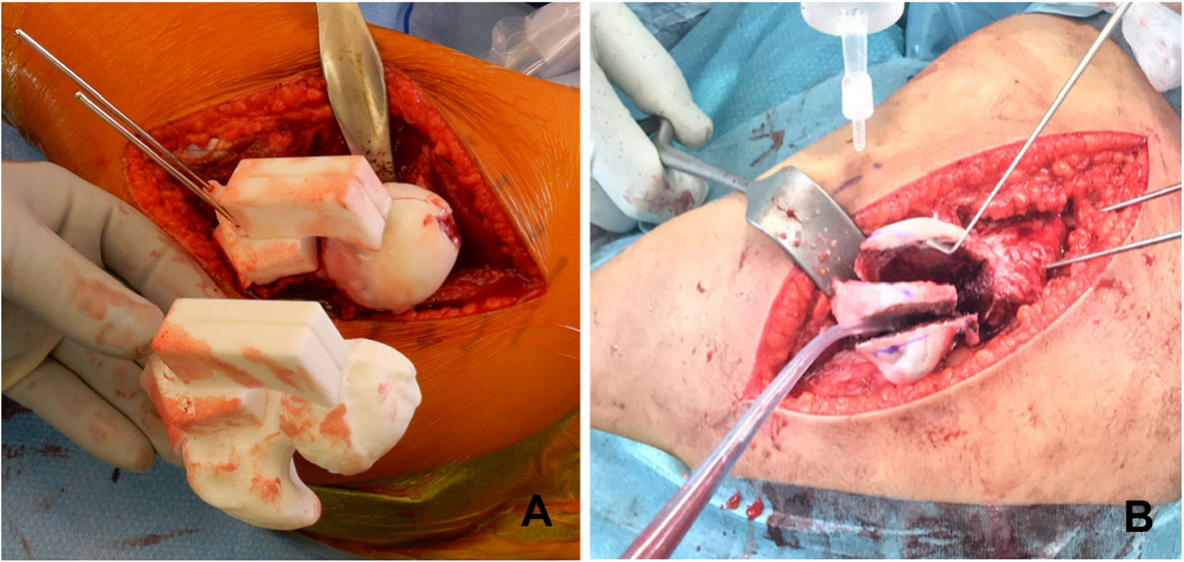
Application of the PSI in the surgery. **a** A patient-specific model of the patient bone was used to verify the correct position of the PSI in the surgery. **b** Resection of the centrally located necrotic fragment

The femoral neck was thereby accessible in its anterior, lateral, and posterior circumference and allowed the positioning of the PSI. Finding the correct position of the PSI is not straight forward and could only be achieved by comparison with a manufactured replica of the patient bone (Fig. 8a). After the fixation of the PSI using two surgical pins of Ø 2.5 mm, the sawing blade [thickness/width/length 1.00/25.00/90.00 mm; Ref. Gomina 265.256.100] was introduced into each of the two cutting slits to perform the medial and lateral head osteotomies under continuous visual control. The level of the subsequent transverse osteotomy at the neck was determined freehand, allowing the necrotic central part and the pedicled lateral fragment to be liberated while the medial part of the head remained stable on the calcar bone (Fig. 8b). After resection of the necrotic part, the mobile fragment was reduced in a freehand fashion, but following the position obtained by the preoperative computer simulation. Under continuous control of the retinacular flap, the fragment could be moved in the cephalad or the caudad direction. It could be shifted posteriorly or anteriorly and could be rotated to finally obtain an optimal surface congruency. The reduced fragment was stabilized with two Ø 3.5 mm cortical screws. The articular step-offs between the contact areas of the fragments were smoothed out using a scalpel in order to restore a transition-free joint surface. The retinaculum and the capsular flap were loosely adapted before the trochanter was reattached.

### Evaluation

The radiological outcome was measured by two independent readers (a fellowship trained radiologist and an orthopedic surgeon) on pre- and postoperative pelvic AP radiographs [19]. For the evaluation of the head shape the ratio of the femur head diameter to the healthy contralateral side [9], the sphericity index [ratio of the minor and major axis of the ellipsoid femoral head] [19], and the Stulberg classification [9] were assessed. The Stulberg classification was measured to evaluate the chances of developing coxarthrosis in patients based on the severity of femoral head deformities, ranging from 1 (normal joint) to 5 [prognosis: severe early arthritis] [9]. For the evaluation of hip containment, the extrusion index (ratio of head extrusion distance and containment) [27], the lateral center-edge angle (LCE), the Tönnis angle [28], and the Shenton line [29] were measured. Additionally, the centrum-collum-diaphyseal (CCD) angle was obtained to assess whether the surgery affected varus or valgus alignments and the preoperative Waldenströem classification [30] for the definition of the disease state. For effort, the evaluation time and costs associated with the new technique were recorded. Radiologic values were tested for normal distribution using the Shapiro-Wilk test and for statistical relevance (*p* ≤ 0.05) using Wilcoxon signed ranks test.

## Results

The mean patient age at the time of surgery was 14 years [age range: 9–18]. All patients had a concomitant acetabular reorientation done by PAO and relative femoral neck lengthening [18]. The mean follow-up time was 17.5 months (±2.5).

The results of the pre- and postoperative radiological assessment are given in Table 1. The extrusion index, LCE angle, and Tönnis angle significantly changed from dysplastic values preoperatively to containing values postoperatively. Changes in the CCD angle were not significant. The Shenton line was only intact in 50% of the patients and could be restored in all patients. The diameter and sphericity indexes also improved significantly after surgery as well as the Stulberg grading. In the latter, pre-operatively 3 patients were categorized class 5, 2 patients as class 4, and one patient as class 3, which improved postoperatively (*p* = 0.026) to 3 patients classified as 2 and 3 patients as 1.

**Table 1:** Pre- and postoperative radiologic assessment measured using AP pelvic X-rays

Radiologic value	Preoperative (avg, ±)	Postoperative (avg, ±)	Difference (avg, ±)	***p***-value (Wilcoxon signed rank test)
**Diameter index (%)**	118.2% (11.21%)	98.2% (2.28%)	20% (10.22%)	0.043
**Sphericity index (%)**	51.49% (10.47%)	72.96% (7.33%)	21.47% (7.21%)	0.028
**Extrusion index (%)**	30.77% (11.75%)	− 14.02% (7.74%)	44.8% (15.9%)	0.028
**LCE angle**	22.33° (6.24°)	48.25° (9.65°)	25.92° (7.6°)	0.028
**Tönnis angle**	14.95° (6.08°)	−2.98° (3.95°)	17.94° (4.59°)	0.028
**Intact Shenton lines**	4/6	6/6	2	0.157
**CCD angle**	134.03° (5.43°)	128.5° (8.05°)	−5.51° (4.08°)	0.028

On average, the preliminary analysis of the case [review of image data, data processing, pre-discussion with surgeon] took 63 min (±48) on average. The time for the preoperative computer simulation took 156 min (±64) while 105 min (±68.5) were spent on PSI design. The time expenses resulted in costs of US$ 2800. The cost of manufacturing the CE-marked PSI and the plastic bone models using additive manufacturing was US$ 600.

Complications were reported in three cases; however, none were directly related to the computer simulation method or the navigation technique. In one case, a femoral neck fracture occurred postoperatively due to insufficient residual neck thickness, which was successfully treated by osteosynthesis in a revision surgery. In the second case, the PSI was incorrectly positioned. This was recognized immediately and the cut direction was corrected. This mistake had no negative influence on the clinical and radiological results. In a third case, the intraoperatively observed necrotic area on the lateral side of the femur was larger than expected, which made the operation no longer feasible.

## Discussion

FHRO has been described as an effective surgical treatment for severe deformities of the femoral head [5, 7, 17–20]. Nevertheless, the procedure remains one of the most technically difficult procedures in hip surgery. One reason is that while the underlying geometrical problem of restoring the head sphericity is highly three-dimensional, state-of-the-art planning still relies on 2D measurements because adequate computer methods are not available. Computer simulations have been proven successful in solving 3D planning problems in various extra- [31, 32] and intra-articular [23, 33, 34] corrective osteotomies. One purpose of this study was to investigate the feasibility of using 3D computer simulation for the preoperative planning of FHRO.

The postoperative radiological evaluation of our study showed a clear improvement compared to the preoperative situation. The sphericity improved postoperatively by 20% and the postoperative head diameter difference was reduced from 18.2% to only 1.8% compared to the healthy contralateral side. Compared to previous studies in which FHRO was performed, based on conventional preoperative planning and without support by surgical navigation [19], our results indicate a better reconstruction of sphericity up to 8%. The post-operative evaluation is a limitation of the study as it was only based on the AP X-ray projections and 2D measurements. The use of a post-operative CT scan would have been preferred for research but since the CT scan would have only been used for evaluation purposes, it was not ethically justified regarding the exposure of radiation for the young patients. The application of low-dose CT [35] is a possible solution for basing future radiological outcome evaluation on post-operative CT. The use of CT-reconstructed 3D models in the post-operative evaluation would allow the application of more precise and powerful methods of measurement which have been developed for outcome evaluation of intra-articular osteotomies [23, 34].

Surgical navigation approaches for the hip have been previously reported for extra-articular femur osteotomies and PAO [36]. In this study, we introduced a new intraarticular approach based on PSI tailored for the surgical treatment of FHRO. The precision of the navigation by PSI is mainly determined by how well the intended position of the PSI on the bone can be reproduced intraoperatively. A previous study [37] showed that for the proximal tibia, PSI malpositioning can result in severe surgical failures such as screw penetration or tibia plateau fracture. Since the footprint of the proximal femur on which the PSI can be placed is very small, we introduced medial and lateral hooks in order to increase stability. Nevertheless, finding the right position for the PSI on the bone remains challenging. A great support tool for the surgeon is the 3D-printed patient-specific bone replica on which the PSI fit perfectly. Despite these precautions, malpositioning occurred only in one case. To prevent this in future cases, we integrated slits in the replica, representing the planned head osteotomies such that better comparison between planning and intraoperative situations for the surgeon is possible.

Another challenge is the difficulty of the assessment of the cartilage quality from CT pictures. In one of our cases, the cartilage destruction was worse than expected and unfavorably distributed, a condition that did not allow the surgeon to proceed with the FHRO as planned. Integrating information about the cartilage condition into the simulation process by recording a preoperative MRI would allow for better planning and can help avoid such unfortunate situations intraoperatively.

Our study and the described technique has several limitations. With 324 min on average, the effort required for pathology analysis, computer simulation, and PSI design is still high. Furthermore, the creation of the preoperative simulation of an FHRO presupposes extensive anatomical and surgical knowledge, thus requiring stronger support from the surgeon when compared to other interventions. However, for a cost-benefit analysis, it has to be considered that the only established treatment option for the young patient population of the study would be total hip arthroplasty (THA). Treating adolescents by THA remains a big compromise as one or more revision surgeries would be required during the patient’s lifetime [38]. Our approach could contribute to further standardization of FHRO which could lead to the procedure becoming attractive for other highly-specialized centers.

Another limitation of this study is the small sample. It has to be highlighted that patients undergoing FHRO have to be selected very carefully depending on various factors. The short follow-up time only allowed for reporting intra-operative experiences and preliminary radiological results. The application of post-operative MARS (metallic artifact reduction sequences) would permit a more comprehensive post-operative assessment, but the study design and setting (prototype study; single cases in different centers) did not allow the implementation. Another drawback is the retrospective fashion of the study. However, it may have created a useful basis for a prospective multicenter project. Our proposed approach causes additional expenses of 3400 USD per patient. However, we think these additional costs can be justified in young patients if the need for total hip arthroplasties can be delayed as long as possible. Another possibility of reducing the costs of our approach and time is the automation of the planning process which is subject to ongoing research. The work of Carrillo et al. [39] has already demonstrated with the example of extra-articular forearm osteotomies that clinically acceptable 3D planning solutions can be achieved through sophisticated automatic computer methods. Another technical improvement would be the integration of patient-specific cartilage models into the computer simulation using image fusion techniques. The findings of this pilot study should also form the base to justify a multi-centric, prospective clinical trial. However, the implementation of such a study remains very difficult due to the small number of individual cases distributed among different centers worldwide.

## Conclusion

In 1999, DiGioia et al. [40] postulated future surgical technologies, including tools capable of simulating each step of surgery with 3D models of the patient anatomy. Through advances in computational power and the development of enabling technologies such as additive manufacturing, their vision has been turned into clinical practice. However, particularly in orthopedics, several complex procedures with a small caseload still exist for which— often due to economic reasons—no computer-based solution has been developed yet. Our study serves as an example of how emerging technologies are increasingly shaping orthopedic surgery towards digital and personalized medicine, even in very complex and rare interventions. The clinical feasibility of our approach has been successfully demonstrated. Nevertheless, the complications and challenges reported in our study indicate that FHRO should only be performed by very experienced surgeons in highly specialized centers.

## Data Availability

The datasets generated and analyzed during the current study are not publicly available due to protection of patient data and characteristics since the number of interventions worldwide is extremely small but are available from the corresponding author on reasonable request.

## Abbreviations

LCP: Legg–Calvé–Perthes
FHRO: Femoral head reduction osteotomy
PAO: Periacetabular osteotomy
PSI: Patient-specific instruments
THA: Total hip arthroplasty
LCE: Lateral center-edge angle
CCD: Centrum-collum-diaphyseal angle
